# The HIV-1 protective -35SNP effect in Caucasians is CD8 T cell mediated

**DOI:** 10.1186/1742-4690-9-S2-P281

**Published:** 2012-09-13

**Authors:** T Corrah, S Brackenridge, N Goonetilleke, H Yang, S Deeks, L Dorrell, M Cohen, A McMichael

**Affiliations:** 1 Addenbrooks Hospital, University of Cambridge, Cambridge, UK; 2 Weatherall Institute of Molecular Medicine, University of Oxford, Oxford, UK; 3 University of California San Francisco, San Francisco, CA, USA; 4 University of North Carolina School of Medicine, NC, USA

## Background

Previous studies in Caucasians have observed that a single nucleotide polymorphism 35kb upstream of the HLA-C gene (-35SNP) associates with control of HIV-1 viral load set-point and cell surface expression of HLA-C. HIV-1 selectively downregulates HLA-A and HLA-B but not HLA-C, via the action of the Nef protein. Thus it has been speculated that higher cell surface HLA-C expression results in a stronger HLA-C-restricted T cell response which might play a role in the control of HIV-1 replication in individuals with the protective -35C variant. However, HLA-C-restricted CD8 T cell responses are relatively weak and we could find no difference in functional HLA-C-restricted CD8 T cell activity measured by IFN-γ ELISPOT assay, according to -35SNP genotype. Therefore, we aimed to examine if there is any correlation between total CD8 T cell function and the -35SNP.

## Methods

The viral suppression assay, which involves directly infecting autologous CD4 T cells with primary HIV-1 strains and co-culturing with autologous CD8 T cells, was used as a surrogate for immune control in vivo. The CD8 T cells from 46 antiretroviral therapy naïve HIV-1 infected Caucasians were assessed using this assay.

## Results

When CD8 T cell antiviral activity was grouped according to -35SNP genotype, the -35CC group possessed significantly higher CD8 T cell antiviral activity than the -35TT group (p=0.0151; Mann-Whitney). Protective HLA-B alleles were always in linkage disequilibrium with HLA-C alleles that are in linkage disequilibrium with the -35C allele. Similarly risk HLA-B alleles were in linkage disequilibrium with HLA-C alleles that are in linkage disequilibrium with the -35T allele.

## Conclusion

In conclusion, the protective -35SNP effect in HIV-1 disease is mediated through CD8 T cells. However, the -35SNP may simply be a marker for protective and risk HLA-B alleles.

